# Families of Molecular Hexa- and Trideca-Metallic Vanadium(III) Phosphonates

**DOI:** 10.3390/ma3010232

**Published:** 2010-01-08

**Authors:** Sumit Khanra, Rachel Shaw, Madeleine Helliwell, Floriana Tuna, Christopher A. Muryn, Eric J. L. McInnes, Richard E. P. Winpenny

**Affiliations:** School of Chemistry, The University of Manchester, Manchester M13 9PL, UK

**Keywords:** polyoxovanadium clusters, vanadium(III), phosphonates

## Abstract

The synthesis and structural characterization of two families of low-valent vanadium(III) {V_6_P_4_} and vanadium(III/IV) {V_13_P_8_} phosphonate complexes are reported. Magnetic characterization is reported for representative examples.

## 1. Introduction

High-valent vanadium(IV/V) (organo)phosphates are an important sub-class of polyoxovanadates with a rich structural chemistry [1]. The chemistry of molecular examples is dominated by sphere- or bowl-like clusters that display rich host-guest chemistry [2,3,4]. Low-valent vanadium(III/IV) phosphonates are much rarer and tend to be extended lattice systems [1,5,6]. Molecular low-valent systems – the vanadium(III/IV) form of the organophosphate-templated polyoxovanadates—are rarer still; until recently Zubieta’s (Ph_4_P)(Bu_4_N)[(V^IV^O)_6_V^III^{BuP(O)_2_OPO_3_}_6_] was the sole example [7]. We recently reported straightforward synthetic routes to several new low-valent compounds, including {V_4_P_4_}, {V_5_P_6_}, {V_6_P_4_}, {V_8_P_8_}, {V_8_P_16_}, {V_9_P_3_} and {V_13_P_8_} examples [8,9]. In this work we expand on two of these families – {V_6_P_4_} and {V_13_P_8_} – to illustrate the generality of this approach.

## 2. Results and Discussion

**Figure 1:**
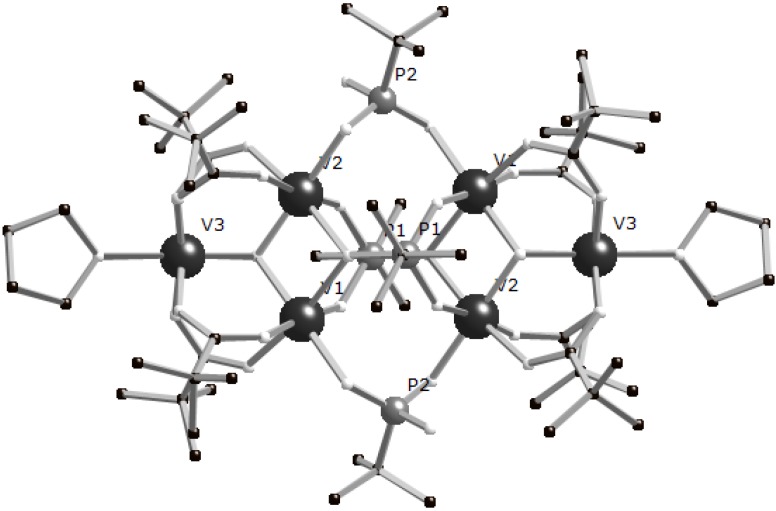
Structure of **2** in the crystal.

In our previous work we showed that, in common with clusters of later transition ions, molecular V(III) phosphonates can be prepared from {M_3_(μ_3_-O)} building blocks. We found that these {V(III)_3_} triangles could be formed *in situ* which is simpler than preparing and isolating the air sensitive [V_3_O(O_2_CR)_6_L_3_]^+^ (L = terminal ligand) basic metal carboxylates. For example, [V^III^_6_(O)_2_(O_2_C^t^Bu)_8_(HO_2_C^t^Bu)_2_(HO_3_P^t^Bu)_2_(O_3_P^t^Bu)_2_] (**1**) is prepared from the one-pot reaction of pivalic acid, VCl_3_ and *t*-butylphosphonate in MeCN with Et_3_N as base [8]. Analogues of **1** can be prepared similarly. If [VCl_3_(thf)_3_] is used in place of VCl_3_ in an otherwise identical reaction then [V^III^_6_(μ_3_-O)_2_(^t^BuPO_3_)_2_(^t^BuPO_3_H)_2_(^t^BuCO_2_)_8_(thf)_2_] (**2**) is formed. **2** is centrosymmetric with two oxo-centred vanadium triangles linked via four phosphonates (Figure 1). The two fully deprotonated RPO_3_^2-^ 1,3-bridge one edge of each triangle, formally replacing one carboxylate in the “parent” [V_3_O(O_2_C^t^Bu)_6_L_3_]^+^ triangle, with the third arm providing a μ_2_-bridge on the second triangle ([4.211]-binding mode in Harris notation [10]). The two singly deprotonated (RPO_3_H)^-^ 1,3-bridge between the triangles, with the two coordinated arms binding terminally ([2.110]-binding mode). The two thf molecules act as terminal ligands at the vanadium ions not involved in linking the two triangles – in complex **1** these are replaced by pivalic acid.

The bridging carboxylates can also be substituted: reaction of [VCl_3_(thf)_3_], PhCO_2_H and ^t^BuPO_3_H_2_ (6:8:4) with Et_3_N in EtOH gives [V^III^_6_(O)_2_(^t^BuPO_3_)_2_(^t^BuPO_3_H)_2_(PhCO_2_)_8_(EtOH)_2_] (**3**). Now eight benzoates span edges of the triangles and alcohol is the terminal ligand. If a higher proportion of phosphonic acid is used in the reaction then phosphonate can also act as the terminal ligand: reaction of [VCl_3_(thf)_3_], ^t^BuCO_2_H and ^t^BuPO_3_H_2_ (6:8:6) with Et_3_N in MeCN gives (Et_3_NH)_2_[V^III^_6_(O)_2_(^t^BuPO_3_)_2_(^t^BuPO_3_H)_2_(^t^BuCO_2_)_8_(^t^BuPO_3_H)_2_] (**4**). Complex **4** has two singly deprotonated (RPO_3_H)^-^ terminal ligands making it a dianion.

**Figure 2:**
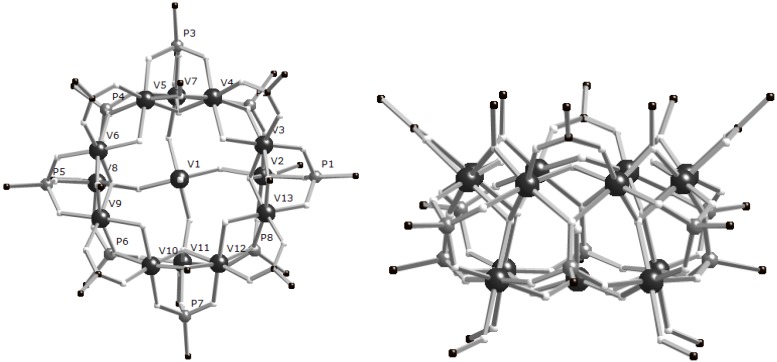
(left) Structure of the cation of **6** in the crystal. (right) “Side-on” view highlighting layered structure. R, R’ and R’’ groups removed for clarity.

**Figure 3:**
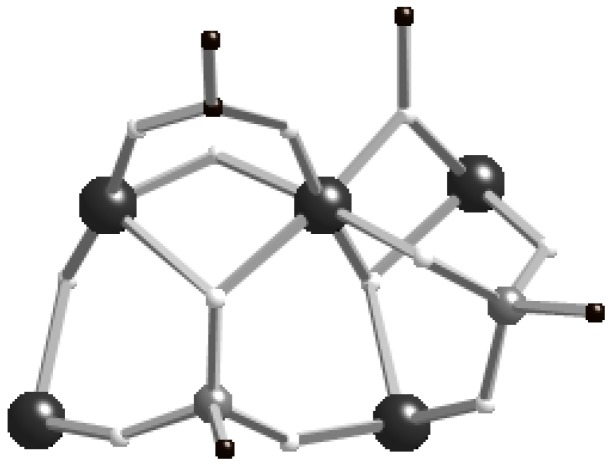
Fragment of structure of **6** highlighting the phosphonate binding modes. The top three vanadium ions are in the “upper” V_8_ ring.

Much larger clusters result if the reactions in alcohols are performed solvothermally. We previously reported [V^III^_12_(V^IV^O)(μ_3_-OH)_4_(μ_2_-OH)_8_(μ_2_-OEt)_4_(EtOH)_4_(PhCO_2_)_4_(O_3_P^t^Bu)_8_]Cl_2_ (**5**) from reaction of [VCl_3_(thf)_3_], PhCO_2_H and^ t^BuPO_3_H (13:8:4) with KOEt in EtOH at 150 °C [8]. As with the hexametallic **1**, we can prepare a number of analogues of **5**, substituting the phosphonate RPO_3_H_2_, carboxylic acid R’CO_2_H and alcohol R”OH to give [V_12_(VO)(*μ_3_*-OH)_4_(*μ_2_*-OH)_8_(*μ_2_*-OR’’)_4_(R’’OH)_4_(R’CO_2_)_4_(RPO_3_)_8_]X_2_ where R = ^t^Bu, R’ = ^t^Bu, R’’ = Et, X_2_ = (OH)Cl (**6**); R = ^t^Bu, R’ = Ph_2_C(H), R’’ = Et, X_2_ = (OH)Cl (**7**); R = PhCH_2_, R’ = ^t^Bu, R’’ = Et, X = Cl (**8**); R = ^t^Bu, R’ = ^t^Bu, R’’ = Me, X_2_ = (OH)Cl (**9**). The structures of **5**–**9** are similar, being based on a square of{V^III^_3_(*μ_3_*-OH)} triangles with a central vanadyl ion (V1 in Figure 2) bound to the center of the cage. The square of triangles is formed so that eight V^III^ centres are in one plane with the remaining four V^III^ ions forming a square above this octagon. Four phosphonates each bind a face of each triangle, each arm binding terminally ([3.111]-binding mode; Figure 3). The other four phosphonates (P2 and P5 and symmetry equivalents in Figure 3b) link the triangles, 1,3-bridging edges of neighbouring triangles with one arm binding to both ([4.211] binding mode, Figure 3). The {VO}^2+^ ion is bridged by four μ_2_-OH to the four V^III^ centers within the square plane. Each of these V^III^ ions has a terminal R’’OH, all of which H-bond to one of the counter-ions. The bridging in the octagonal plane alternates between a [3.111]-phosphonate with an alkoxide and a μ_3_-OH, and a [4.211]-phosphonate with a μ_2_-carboxylate and a μ_2_-OH. The four hydroxides lie towards the centre of the cavity. The central vanadium ion binds the only terminal oxide in the structure and is in the +4 oxidation state. All other metal ions in the complexes are in the +3 oxidation state. 

**Figure 4:**
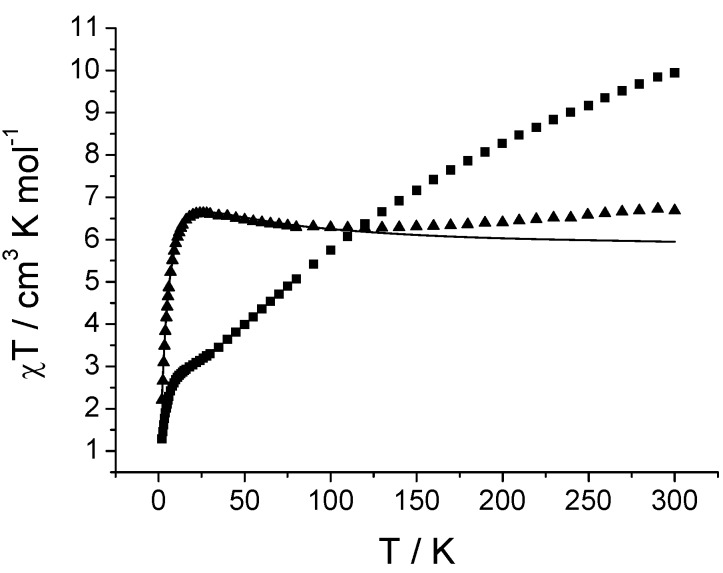
Magnetic data for **3** (triangles) and **5** (squares) and calculated curve for **3** (solid line) using the values in the text.

**Figure 5:**
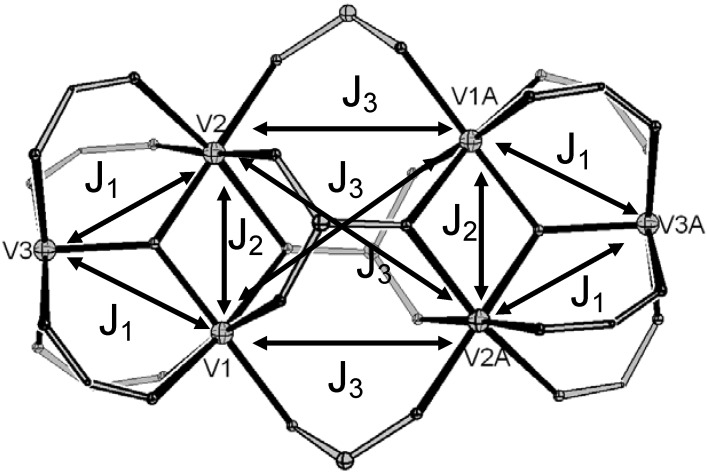
Model for exchange coupling in complex **3**.

We have undertaken magnetic studies on two representative examples of the hexa- and trideca-metallic families, **3** and **5**. The room temperature *χ*_M_*T* value of **3 **is 6.5 cm^3^ K mol^−1^; assuming we can treat the V^III^, d^2^ ion as *s* = 1 we would expect 6.0 cm^3^ K mol^−1^ for six non-interacting ions with *g* = 2.0. *χ*_M_*T* decreases slightly on cooling and then increases slowly to a maximum at ca. 25 K before collapsing to 2.1 cm^3^ K mol^−1^ at 2 K (Figure 4). We have attempted to model this behaviour. The simplest meaningful model has three unique exchange interactions [Figure 5, Hamiltonian (1)]: (i) the carboxylate-bridged edges within each {V_3_(μ_3_-O)} unit, *J*_1_ (ii) the unique, phosphonate-bridged edge with each triangle, *J*_2_; (iii) between vanadium ions in different triangles, bridged by 1,3-phophonates, *J*_3_. 


H = - 2*J_1_*[S_1_.S_3_+S_2_.S_3_+S_1A_.S_3A_+S_2A_.S_3A_] - 2*J_2_*[S_1_.S_2_+S_1A_.S_2A_] - 2*J_3_*[S_1_.S_2A_+S_1A_.S_2_+S_1_.S_1A_+S_2_.S_2A_] (1)

A reasonable, but not good, fit is found with *J*_1_ = +7.2, *J*_2_ = −4.5 and* J*_3_ = −0.95 cm^−1^ with *g* = 1.95. The weak exchange interactions are not surprising given precedent in the literature from dimers and trimers with related bridging motifs [9]. These *J*-values can be interpreted as giving an *S* = 3 ground state for each triangle which are then antiferromagnetically coupled to each other. This gives an *S* = 0 ground state but with many low-lying excited states; this is consistent with low temperature magnetization (*M*) *vs*. applied magnetic field (*H*) which fail to saturate up to 7 T and 1.8 K.

**Figure 6:**
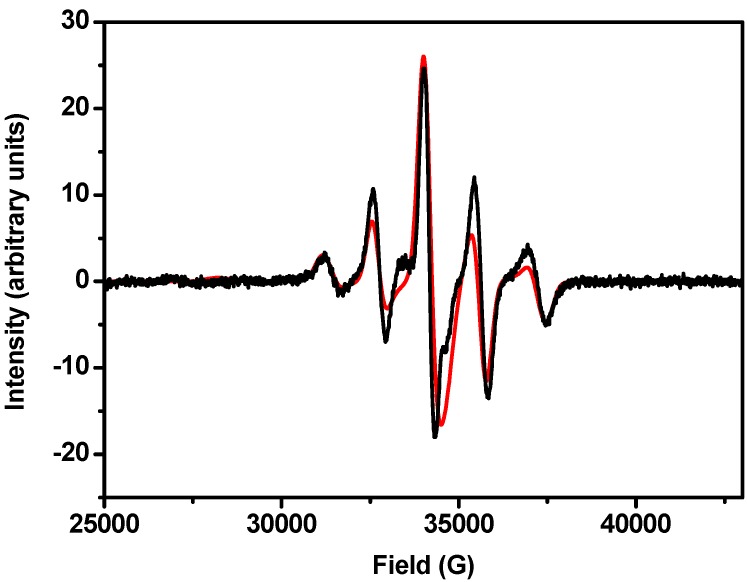
W-band EPR spectrum of a polycrystalline sample of **5** (black) and simulation (red) with *S* = 5/2, *D* = −0.14 cm^-1^ and *g* = 1.96.

Complex **5** has a room temperature *χ*_M_*T* value of 9.94 cm^3^ K mol^−1 ^and is already decreasing rapidly with decreasing temperature (Figure 4). *χ*_M_*T* plateaus in the 20–30 K region at ca. 3.7 cm^3^ K mol^−1^. We have not attempted to model this behaviour, but we can show that it is consistent with the odd-integer electron count arising from {V^III^_12_(V^IV^O)}: a low temperature W-band (94 GHz) EPR spectrum of **5 **is characteristic of an *S* = 5/2 ground state for the cluster (Figure 6). This would be expected to give rise to a low temperature limiting *χ*_M_*T* value of 4.2 cm^3^ K mol^−1^ for *g* = 1.95.

## 3. Experimental Section 

All manipulations were conducted under anaerobic conditions (dinitrogen purged glove box and Schlenk line). Solvents were dried before using. All the vanadium compounds are very sensitive to moisture and air. 

*[V^III^_6_(μ_3_-O)_2_(^t^BuPO_3_)_2_(^t^BuPO_3_H)_2_(^t^BuCO_2_)_8_(thf)_2_]* **(2)**: [VCl_3_(thf)_3_] (0.37 g, 1 mmol) was added to a solution of ^t^BuPO_3_H_2_ (0.09 g, 0.66 mmol) in MeCN (10 mL). ^t^BuCO_2_Na (0.16 g, 1.32 mmol) was added to the resultant suspension and the mixture stirred for 24 h and filtered. Green crystals suitable for X-ray analysis were obtained in three weeks (11%). Elemental analysis calcd (%) for C_64_H_126_O_32_P_4_V_6_: C 41.83, H 6.91, P 6.74, V 16.64; found: C 40.94, H 6.7, P 6.38, V 15.4.

*[V^III^_6_(O)_2_(^t^BuPO_3_)_2_(^t^BuPO_3_H)_2_(PhCO_2_)_8_(EtOH)_2_]* **(3)**: [VCl_3_(thf)_3_] (0.37 g, 1 mmol) was added to a solution of ^t^BuPO_3_H_2_ (0.09 g, 0.66 mmol) in EtOH (10 mL). PhCO_2_H (0.16 g, 1.32 mmol) and Et_3_N (0.3 mL, 2.3 mmol) were added to the resultant suspension, and the mixture stirred for 24 h and filtered. Green crystals were obtained in two-to-three weeks (16%). Elemental analysis calcd (%) for C_76_H_90_O_32_P_4_V_6_: C 46.93, H 4.66, P 6.37, V 15.71; found: C 46.58, H 4.55, P 6.08, V 14.82.

*(Et_3_NH)_2_[V^III^_6_(O)_2_(^t^BuPO_3_)_2_(^t^BuPO_3_H)_2_(^t^BuCO_2_)_8_(^t^BuPO_3_H)_2_]* **(4)**: [VCl_3_(thf)_3_] (0.185 g, 0.5 mmol) was added to ^t^BuPO_3_H_2_ (0.069 g, 0.5 mmol) in MeCN (10 mL). ^t^BuCO_2_H (0.07 g, 0.66 mmol) and Et_3_N (0.17 mL, 1.33 mmol) were added and the mixture stirred for 24 h and filtered. Green crystals were obtained after two weeks (20%). Elemental analysis calcd (%) for C_76_H_162_N_2_O_36_P_6_V_6_: C 42.03, H 7.52, N 1.29, P 8.56, V 14.07; found: C 42.53, H 7.67, N 1.31, P 8.23, V 13.35.

*[V_12_(VO)(μ_3_-OH)_4_(μ_2_-OH)_8_(μ_2_-OEt)_4_(EtOH)_4_(^t^BuCO_2_)_4_(^t^BuPO_3_)_8_](OH)Cl* **(6)**: [VCl_3_(thf)_3_] (0.3 g, 0.8 mmol), ^t^BuPO_3_H_2_ (0.069 g, 0.5 mmol), ^t^BuCO_2_H (0.026 g, 0.25 mmol) and KOEt (0.10 g, 1.3 mmol) in EtOH (14 mL) were heated at 150 °C in a sealed Teflon-lined autoclave for 12 h then cooled to give an insoluble solid under a green solution, which was filtered. Green crystals grew from the filtrate after two weeks (36%). Elemental analysis calcd (%) for C_68_H_165_V_13_O_54_P_8_Cl_1_·1.5EtOH: C 29.7, H 6.11, P 8.87, Cl 1.24, V 23.08; found: C 29.47, H 5.96, P 8.58, Cl 1.15, V 21.8

Compounds **7**–**9** were synthesized by analogous reactions. **7**: Yield 30%. Elemental analysis calcd (%) for C_104_H_173_V_13_O_54_P_8_Cl_1_·2EtOH: C 39.01, H 5.68, P 7.45, Cl 1.07, V 19.91; found: C 39.71, H 5.85, P 7.14, Cl 1.1, V 18.59. **8**: Yield 36%. Elemental analysis calcd (%) for C_100_H_132_V_13_O_53_P_8_Cl_2_: C 37.96, H 4.21, P 7.83, Cl 2.24, V 20.94; found: C 37.71, H 4.08, P 7.64, Cl 2.14, V 19.63. **9**: Yield 35%. Elemental analysis calcd (%) for C_62_H_149_V_13_O_54_P_8_Cl_1_: C 26.89, H 5.6, P 9.24, V 24.71; found: C 27.12, H 5.78, P 8.98, V 23.4.

X-ray data for compounds **2–4** and **9** were collected on an Oxford Instruments CCD diffractometer (Mo K_α_, λ= 0.71073 Å), and data for **6**–**8 **on a on Bruker SMART CCD diffractometer using synchrotron radiation (λ=0.67090 Å and 0.69260 Å). In all cases the selected crystals were mounted on the tip of a glass pin by using Paratone-N oil and placed in the cold flow produced by an Oxford Cryo-cooling device. Complete hemispheres of data were collected using ω scans (0.3°, 30–50 s/frame). Integrated intensities were obtained with SAINT+, and they were corrected for absorption using SADABS. Structure solution and refinement was performed with the SHELX package [11]. The structures were solved by direct methods and completed by iterative cycles of ∇F syntheses and full-matrix least-squares refinement against F^2^. Crystal data and refinement parameters are given in Table 1 and Table 2. Cif files are in electronic supplementary information. CCDC deposition numbers 760561 – 760567.

**Table 1:** Crystal data for 2–4.

Compound	2	3	4
formula	C_64_H_126_V_6_O_32_P_4_	C_79_H_104_V_6_O_36_P_4_	C_76_H_162_V_6_N_2_O_36_P_6_
*M*	1837.17	2059.14	2171.54
cryst syst	orthorhombic	monoclinic	monoclinic
space group	*Pbca*	*P*2_1_/*n*	*P*2_1_/*n*
*a*/Å	21.2682(11)	17.5267(14)	14.7996(16)
*b/*Å	19.6989(10)	14.7323(13)	21.810(3)
*c*/Å	22.0589(11)	20.2027(17)	17.6498(17)
α/deg	90	90	90
β/deg	90	103.887(8)	107.009(12)
γ/deg	90	90	90
*U*/Å^3^	9241.8(8)	5064.0(7)	5447.9(10)
*T*/K	100(2)	100(2)	100(2)
*Z*	4	2	2
μ/mm^-1^	1.320	1.350	1.324
unique data	8174	5138	4255
data with *F*_o_ > 4σ (*F*_o_)	7011	2003	2430
R1, wR2*^a^*	0.0553, 0.1642	0.0711, 0.1524	0.0955, 0.2177

**Table 2:** Crystal data for 6–9.

Compound	6	7	8	9
formula	C_68.5_H_168.75_V_13_Cl_1_O_56_P_8_	C_108_H_184_V_13_Cl_1_O_56_P_8_	C_100_H_134_V_13_Cl_2_O_55_P_8_	C_62.75_H_162_V_13_Cl_1_O_57.75_P_8_
*M*	2834.21	3323.98	3196.96	2786.35
cryst syst	orthorhombic	tetragonal	monoclinic	monoclinic
space group	Aba2	*P4/n*	*C2/c*	*C2/c*
*a*/Å	50.480(10)	21.229(5)	28.2520(13)	55.477(2)
*b/*Å	34.466(9)	21.229(5)	26.0681(13)	16.4228(3)
*c*/Å	34.340(9)	18.408(5)	20.1169(10)	29.2038(8)
α/deg	90	90	90	90
β/deg	90	90	93.5800(10)	93.861(3)
γ/deg	90	90	90	90
*U*/Å^3^	59746(25)	8296(3)	14786.7(12)	26546.9(14)
*T*/K	150(2)	150(2)	150(2)	100(2)
*Z*	16	2	8	8
μ/mm^-1^	1.260	1.331	1.436	1.394
unique data	53420	7338	16284	20725
data with *F*_o_ > 4σ (*F*_o_)	42216	4641	12222	11195
R1, wR2*^a^*	0.0671, 0.1742	0.0879, 0.2685	0.0626, 0.1859	0.0802, 0.2156

## 4. Conclusions 

From this work, and our previous work in this area, it is becoming apparent that V(III) chemistry with phosphonates has a good deal in common with Fe(III) chemistry [12]—the major difference being the high air-sensitivity of the vanadium(III) oxidation state. By subtle variations of carboxylate and phosphonate we can make two large families of cages - {V_6_P_4_} and {V_13_P_8_} - and we believe further families should also be accessible by further variation. Thus far the magnetic properties have been disappointing, but this is often the case with early investigations of new families of polymetallic compounds.
